# Incessant Atrial Tachycardia: Problem Solving With Minimally Invasive Surgery

**DOI:** 10.7759/cureus.18643

**Published:** 2021-10-10

**Authors:** Pedro Queirós, Gualter Silva, João Almeida, Daniel Martins, João Primo

**Affiliations:** 1 Cardiology, Centro Hospitalar de Vila Nova de Gaia/Espinho, Vila Nova de Gaia, PRT; 2 Electrophysiology, Centro Hospitalar de Vila Nova de Gaia/Espinho, Vila Nova de Gaia, PRT; 3 Cardiothoracic Surgery, Centro Hospitalar de Vila Nova de Gaia/Espinho, Vila Nova de Gaia, PRT

**Keywords:** tachycardia induced cardiomyopathy, left atrial ablation, atrial tachycardia, interventional cardiology, video-assisted thoracoscopic surgery (vats), minimally invasive surgery, radio-frequency ablation

## Abstract

A 35-year-old female with sarcoidosis sought medical attention due to palpitations. The ECG showed an atrial tachycardia (AT), apparently originating in the left atrium. A 24-hour Holter monitoring revealed AT to be present during the entire day. Cardiac magnetic resonance exhibited no cardiac involvement by sarcoidosis but registered a mildly depressed left ventricular ejection fraction (LVEF). Atrial electroanatomical mapping showed the earliest activation zone on the distal portion of the left atrial appendage (LAA). Considering the high risk for perforation with catheter ablation in this region, she was sent to thoracoscopic surgical LAA exclusion with a clip device; it was possible to witness the termination of the arrhythmia during the procedure. She was safely discharged two days after surgery and has completed a one-year follow-up without recurrence of AT or symptoms, and with normalization of LVEF.

## Introduction

Atrial tachycardia (AT) is a relatively rare condition, accounting for approximately 10-15% of patients referred to catheter ablation of supraventricular tachycardias. Although generally a benign disease, up to 30% of patients can present with frequent runs or incessant tachycardia, with some even developing tachycardia-induced cardiomyopathy [1]⁠. This arrhythmia is frequently refractory to medical therapy while having a great response to catheter ablation, which has become the standard of care [2]⁠. However, while this technique is generally safe, there is a risk, albeit very small, of severe complications, namely myocardial rupture with cardiac tamponade or thromboembolism. Also, this risk seems to increase when the ablation is done in the left atrial appendage (LAA), a location that is particularly thin-walled, highly trabeculated, and prone to thrombus formation [3,4]⁠. Thus, alternative therapies are important for patients in this situation. We describe the case of a young female patient with an incessant AT originating in the LAA, deemed unsuitable for catheter ablation and successfully treated with a minimally invasive thoracic surgery.

## Case presentation

Patient presentation

A 35-year-old female was admitted to a hospital with complaints of dyspnea and dry cough. During the diagnostic work-up, she was diagnosed with pulmonary sarcoidosis and was later discharged with a daily dose of prednisone. During her follow-up at that hospital, she also complained of persistent palpitations, aggravated during her daily activity but present throughout the entire day, and was initially diagnosed with inappropriate sinus tachycardia, assumed to be related to her decompensated sarcoidosis. She was started on ivabradine, and the palpitations improved; her respiratory illness also improved, as her sarcoidosis was now treated and under control.

A few months later, after changing her residence to another city, the palpitations once again aggravated. She then sought medical attention at our hospital. Physical examination was unremarkable at the time of the visit, and the patient noted her intention to become pregnant.

Initial workup

**Figure 1:**
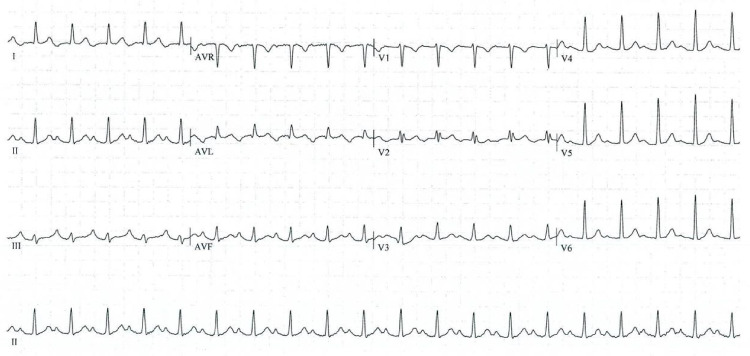
Patient's first ECG during initial workup The negative P waves in leads I and aVL, together with their positivity in the inferior leads, are highly suggestive of an origin in the left atrial appendage.

As the patient had persistent palpitations, a 12-lead ECG was acquired to diagnose the arrhythmia. The ECG showed an AT with a heart rate of 120bpm, apparently originating in the left atrium; the P wave was positive in V1, negative in lead I and aVL, and positive in the remaining leads (Figure 1). No other significant changes were noted. A 24-hour Holter monitoring showed AT during the entire day, suggesting a high burden of arrhythmia.

A transthoracic echocardiogram was performed, showing no structural cardiac disease, with preserved atrial dimensions and biventricular systolic and diastolic function. Cardiac involvement by sarcoidosis was considered, so a cardiac magnetic resonance was done, which showed no cardiac involvement by the disease but showed a depressed left ventricular ejection fraction (LVEF) of approximately 39%. The remaining examination was unremarkable. Chest x-ray showed no signs of a flared pulmonary sarcoidosis; in fact, the exam was grossly unremarkable. Basic lab workup was also normal.

Diagnosis and management

**Figure 2:**
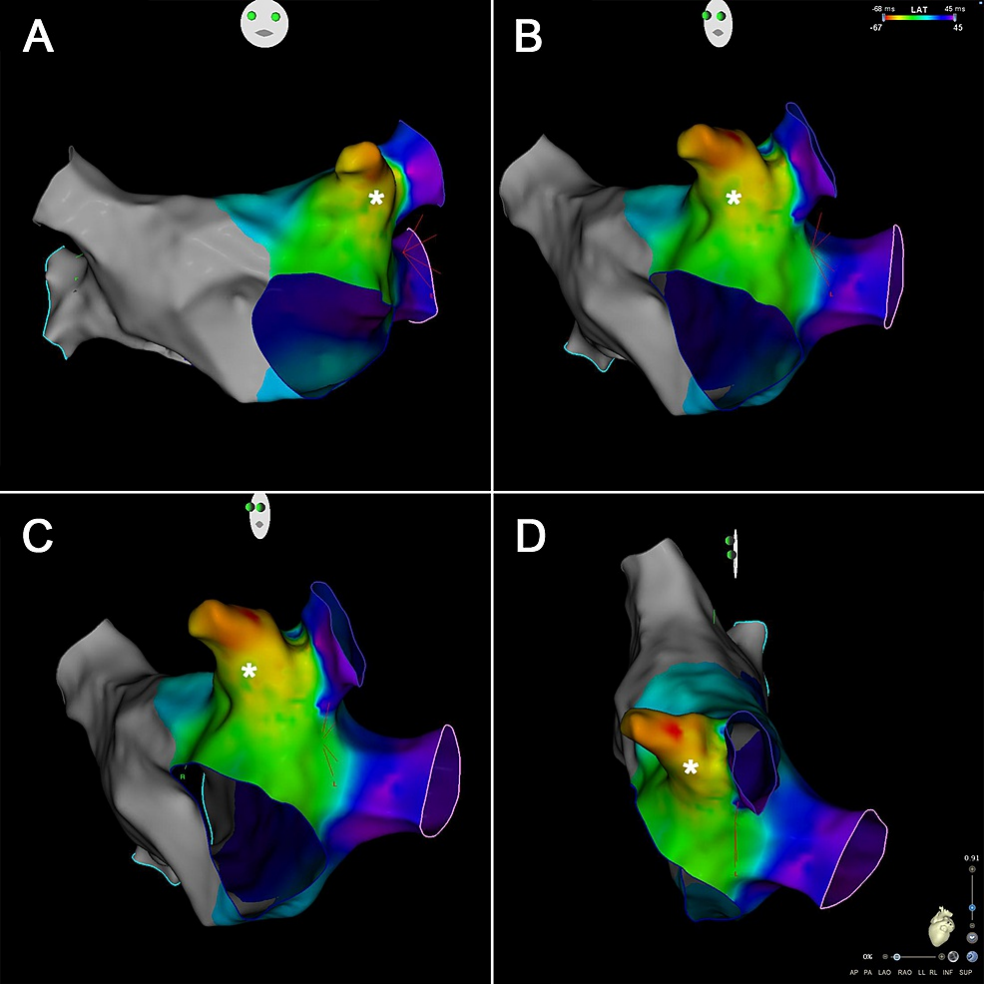
Electroanatomical mapping of the left atria Multiple angles showing the activation latency of the left atria produced by the atrial tachycardia. The color gradient shows the time to activation, with red being the earliest and thus corresponding to the origin of the tachycardia, located near the tip of the left atrial appendage (marked with an asterisk). Panel A shows the mapping from an anterior view, B and C from differently angled left lateral views, and D from a regular left lateral view.

Taking into consideration the findings during the initial workup, we made the diagnosis of incessant AT, possibly resulting in tachycardia-induced cardiomyopathy. According to the most recently published scientific society recommendations and available evidence, we proposed the patient undergo catheter ablation. However, taking into account the patient's preference, a trial of medical therapy was first started with a beta-blocker, but without success. Also, the use of other antiarrhythmic drugs, namely calcium channel blockers, class IC drugs, and amiodarone, was not considered given the presence of a depressed LVEF and the intention of the patient to get pregnant. We then decided to proceed to catheter ablation. Electroanatomical mapping of the left atrium showed the earliest activation during tachycardia to be in the tip of the left atrial appendage (LAA), at least 15mm from its neck (Figure 2). During catheter manipulation inside the LAA, interruption of the tachycardia through mechanical stimulation was observed. However, we decided not to proceed with the ablation, as the thin-walled and highly trabeculated extremity of the appendage is a location particularly prone to perforation during the procedure, which could result in cardiac tamponade.

**Figure 3:**
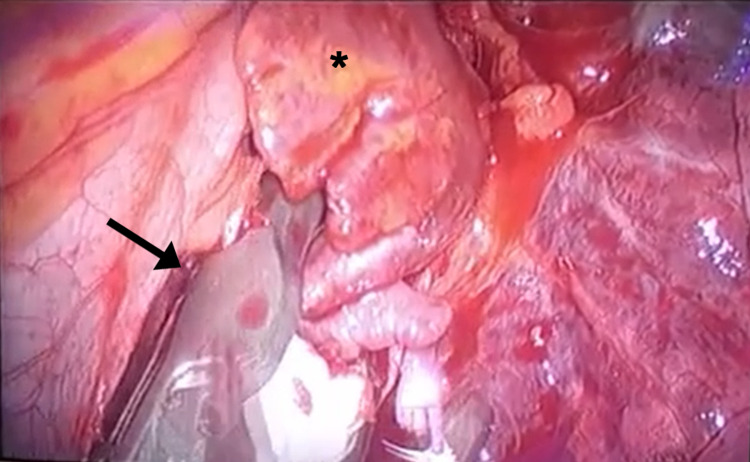
Left atrial appendage clipping as seen by thoracoscopy Surgeon's view during video-assisted thoracoscopic surgery. The clip (arrow) is being deployed in the neck of the left atrial appendage (asterisk), in order to isolate it from the rest of the left atrium.

After the failure of conventional therapy, we discussed the possibility to undergo surgical exclusion of the LAA with the patient and the cardiac surgeon. The procedure was considered feasible with a minimally invasive approach, and would result only in a very short hospital stay; the patient underwent a left uniportal video-assisted thoracoscopic surgery (VATS), with LAA exclusion done with a clipping device. During the procedure, it was possible to witness the termination of the atrial arrhythmia during manipulation of the LAA, as well as the absence of contraction after the successful exclusion, suggesting electrical isolation of the LAA (Figure 3, Video 1).

**Video 1:** Clipping of the atrial appendage as viewed by the surgeon

Follow-up

**Figure 4:**
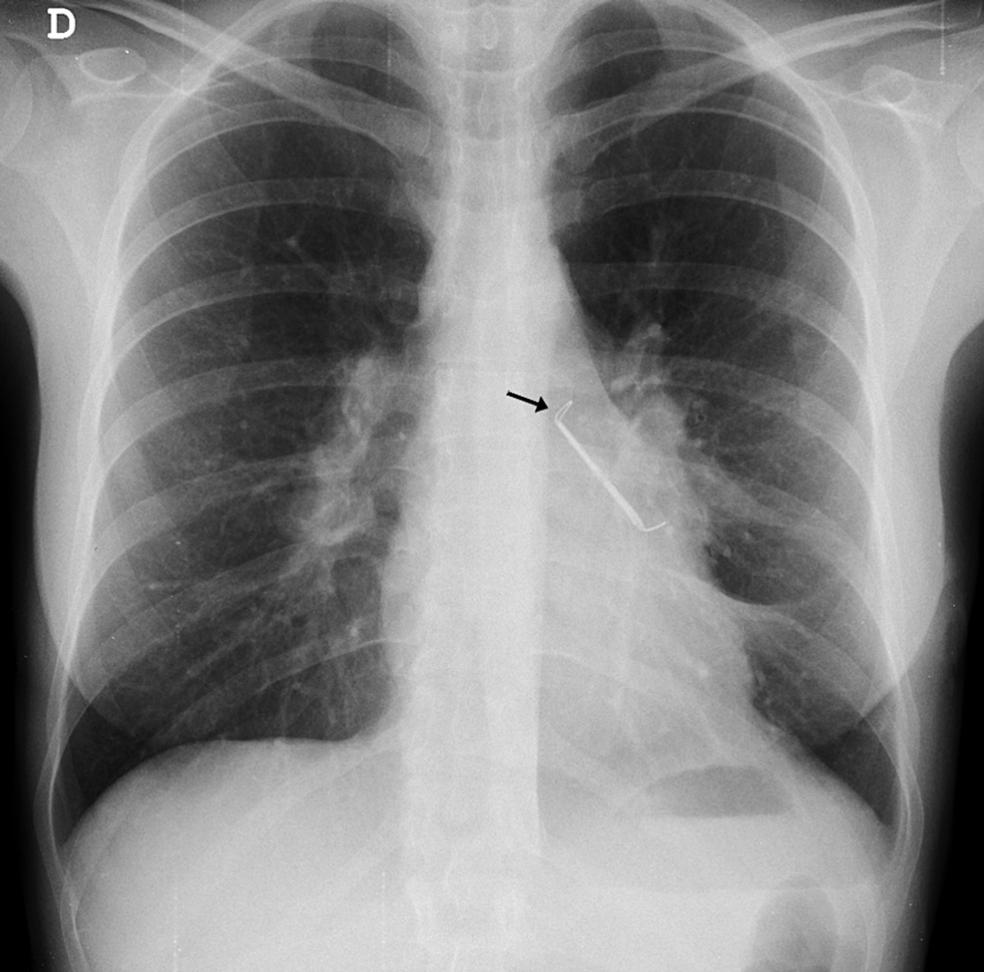
Follow-up chest x-ray the day after surgery The atrial clip (arrow) can be seen in the correct position, and there are no signs of complications.

During the postoperative period, no significant complications were witnessed. On the first day after surgery, a control chest x-ray showed a correctly positioned LAA exclusion clip, with no signs of complications (Figure 4). The patient remained in sinus rhythm after the procedure and was discharged safely and without symptoms in two days. The patient has thus far completed a one-year follow-up as an outpatient after surgery, with no evidence of AT or new-onset symptoms, and normalization of left ventricular systolic function.

## Discussion

We describe a case of an incessant AT originating in the LAA, a difficult location for catheter ablation, successfully treated with minimally invasive surgery. The prevalence of AT in patients under 50 years of age with symptomatic arrhythmia is low (0.46%) [5]⁠, making it one of the least common types of supraventricular tachycardia. Moreover, the LAA is an uncommon site of origin for AT, accounting for 3% to 7.7% of cases, depending on the series [6,7]⁠. In the ECG, the hallmark findings are deep, negative P waves in leads I and aVL, with positivity in the inferior leads, as shown in our case. Indeed, it has been shown that P wave morphology analysis can be used to roughly determine the origin of the AT, with high sensitivity and specificity; as expected, it underperforms when trying to distinguish foci that are too close together [8]⁠, a job best performed by invasive electroanatomical mapping. However, it remains the cornerstone of the initial patient workup and diagnosis and can be used to foresee potential locations that may pose a challenge to catheter ablation.

The recommended form of therapy for AT is currently catheter ablation, even more so when there is concurrent tachycardiomyopathy. This is usually an effective and safe procedure, with success rates reported between 75-100% and complications only occurring in 1.4% [2]⁠. However, two caveats are relevant: first, different AT origins with distinct anatomical characteristics make for different complications and risks; for instance, ablation of an AT with an origin near the atrioventricular node can lead to AV block, whilst the risk of this complication is mostly negligible in other locations. Second, although the risk for complications is generally low, these represent potentially life-threatening and highly morbid events, namely cardiac tamponade, stroke, or need for a permanent pacemaker. These concerns are especially relevant for AT originating in the LAA, a location with a known increased risk of cardiac tamponade and systemic thromboembolism [3,4]⁠, which could offset the benefits of a typically safe procedure. The next treatment option is medical therapy, with most consensus gathered around beta-blockers and calcium channel blockers, given their low risk of side effects; other antiarrhythmic drugs, like class IC drugs, ivabradine, and amiodarone, also present viable treatment options, but with a less favorable risk profile [9]⁠. In our case, we considered the risk of catheter ablation to be not acceptable; we also tried a trial of beta-blocker with no success and deemed the remaining antiarrhythmic drugs to be unsuitable, given the young age of the patient, the depressed LVEF, and the intention to get pregnant. Thus, we were forced to consider different treatment options.

Surgical exclusion of the LAA is often performed to reduce the risk of systemic thromboembolism in patients with atrial fibrillation; however, this procedure is also capable of electrically isolating the LAA. There are few descriptions of surgical exclusion of the LAA as a treatment for AT [10-12]⁠, and most of them are in the pediatric population; all of the reported cases showed termination of the tachycardia and no recurrence during follow-up. The use of uniportal VATS allows for a low risk of complications and short hospital stay, whilst not incurring the risks that could exist with catheter ablation; in fact, it even eliminates the risk of embolic events originating in the LAA. In our view, the minimally invasive surgical treatment option represents a viable alternative to catheter ablation and should not be seen as a last resort to treat AT originating in the LAA.

## Conclusions

Although a rare condition, AT originating in the LAA may present significant treatment challenges. We illustrate how a surgical treatment using a minimally invasive technique can result in termination of the AT, providing a solution to the patient’s illness while carrying a low risk of complications, short hospital stay, and low morbidity. Whilst the existing body of evidence to promote it is small, minimally invasive surgery with LAA clipping seems to be a viable option for patients with AT originating in the LAA and unable to undergo catheter ablation or receive medical therapy.
